# Cancer Omics in Africa: Present and Prospects

**DOI:** 10.3389/fonc.2020.606428

**Published:** 2020-12-14

**Authors:** Islam El Jaddaoui, Imane Allali, Sofia Sehli, Karim Ouldim, Salsabil Hamdi, Najib Al Idrissi, Chakib Nejjari, Saaïd Amzazi, Youssef Bakri, Hassan Ghazal

**Affiliations:** ^1^ Laboratory of Human Pathologies Biology, Department of Biology, Faculty of Sciences, and Genomic Center of Human Pathologies, Faculty of Medicine and Pharmacy, University Mohammed V, Rabat, Morocco; ^2^ Department of Fundamental Sciences, School of Medicine, Mohammed VI University of Health Sciences, Casablanca, Morocco; ^3^ Cancer Research Institute, Fes, Morocco; ^4^ Environmental Health Laboratory, Pasteur Institute, Casablanca, Morocco; ^5^ Department of Surgery, School of Medicine, Mohammed VI University of Health Sciences, Casablanca, Morocco; ^6^ Department of Medicine, School of Medicine, Mohammed VI University of Health Sciences, Casablanca, Morocco; ^7^ National Center for Scientific and Technical Research, Rabat, Morocco

**Keywords:** multi omics, cancer genomics, epigenomics, transcriptomics, metabolomics, proteomics, metagenomics, African continent

## Abstract

During the last century, cancer biology has been arguably one of the most investigated research ﬁelds. To gain deeper insight into cancer mechanisms, scientists have been attempting to integrate multi omics data in cancer research. Cancer genomics, transcriptomics, metabolomics, proteomics, and metagenomics are the main multi omics strategies used currently in the diagnosis, prognosis, treatment, and biomarker discovery in cancer. In this review, we describe the use of different multi omics strategies in cancer research in the African continent and discuss the main challenges facing the implementation of these approaches in African countries such as the lack of training programs in bioinformatics in general and omics strategies in particular and suggest paths to address deficiencies. As a way forward, we advocate for the establishment of an “African Cancer Genomics Consortium” to promote intracontinental collaborative projects and enhance engagement in research activities that address indigenous aspects for cancer precision medicine.

## Introduction

Cancer is essentially a multifactorial disease triggered by the interaction of multiple genes and numerous factors namely age, lifestyle, environmental toxins, and genetic syndromes (1). Cancer is also defined by a subset of abnormal cell clones that develop out of control and can infiltrate and metastasize towards distant organs beyond normal tissue borders (2). As cancer research has entered the precision medicine era, non-molecular characteristics have turned inadequate whilst the use of molecular characteristics is a progressively common research direction. Biomedical researchers aimed for implementing multi omics data in order to obtain new insight into cancer growth and development (3). “Omics” sciences including transcriptomics, genomics, metabolomics, proteomics, metagenomics, and epigenomics include several implementations and aim to significantly enhance our knowledge of cancer growth and progression processes (4). These omics approaches represent an essential part in influencing diagnosis, prognosis, and patients’ treatment (4, 5). Additionally, they are naturally appropriate and very promising for the discovery of useful biomarkers (4). In the multi omics framework, the use of integrative methods became important for gaining more insight into oncological phenomena and step towards the pattern of precision medicine (6).

Considering the enormous areas covered by developed-world advances in molecular and omics-based technologies, the adoption and implementation of these approaches in developed countries yet remain uncertain (7). Cancer is a widespread problem in African countries by dint of ageing and population growth, and increased prevalence of risk factors (8). Europe presents 23.4% of all cancer cases and 20.3% of cancer deaths, pursued by the Americas with 21% of cases and 14.4% of deaths worldwide. Unlike other regions, cancer mortality rates in Asia (57.3%) and Africa (7.3%) are higher than incidence rates (48.4 and 5.8%, respectively) due to the different distribution of cancer types and higher case mortality rates in these areas (9). In 2008, it is estimated that there were 715,000 new cancer cases and 542,000 deaths in Africa (10). The African population is expected to rise by 60 percent overall between 2010 and 2030 and by 90 percent for those 60 and older, the age at which cancer occurs most commonly, as per the United Nations population projections (8). However, facing this rising burden, cancer keeps receiving a relatively low public health priority in Africa, with few exceptions (8).

The International Cancer Genome Consortium (ICGC) was created to support large-scale genome studies regarding tumor cancer from 50 diverse forms and/or subtypes of cancer. It enables systematic studies at the genomic, epigenomic and transcriptomic levels of more than 25,000 cancer genomes (11). Many countries in America, Europe, and Asia are involved in this international project, but African countries shine by their absence. So as not to leave Africa behind in all these highly advantageous developments, there is an urgent need for creativity and maximization of existing infrastructure (7). In this study, we provide past and existing implementations of various multi omics strategies in the African continent’s cancer research sector and address the key challenges regarding the development of these approaches in Africa such as the lack of training programs in bioinformatics in general and omics strategies in particular. Paths forward to address deficiencies will be suggested.

## Cancer Genomics

Valuable new pieces of information about genomic drivers of cancer onset and progression across several anatomical locations have been highlighted thanks to the application of next-generation sequencing (NGS) techniques to discovery projects on large-scale cancer genomics (12). Unlike traditional Sanger sequencing, the NGS has the ability to sequence, very efficiently and at high throughput, gigabases of DNA (13). The majority of NGS approaches rely on DNA template preparation, sequencing and imaging, and data analysis. To prepare the template, current techniques involve randomly splitting the genomic DNA into smaller sizes. The generated template is then attached or immobilized to a rigid support or surface. Thousands to billions of sequencing reactions can occur concurrently due to the immobilization of spatially detached template sites. Because the majority of imaging systems are unable to reveal the fluorescent events, the amplified templates are needed to boost the intensity of sequencing signals (2). NGS can be used to detect small deletions and insertions, loss of heterozygosity in tumor DNA samples, sequence mutations, structural rearrangements, and copy number alterations (12). Due to NGS, beyond the genomic sequencing, which was the initial development objective and application, emerging applications and fields in medicine and biology are becoming a reality. The NGS provides new and fast methods for genome-wide characterization and proﬁling of transcription factor regions, small RNAs, mRNAs, DNA methylation patterns and structure of chromatin, microbiology, and metagenomics (14).

Given that cancer is a genetic disease, sequencing the patient’s genome will allow detecting recurring alterations. Up to now, sequencing of more than 80 forms of cancer worldwide has been achieved. Most prominent actors are the Cancer Genome Atlas (TCGA) project and the International Cancer Genome Consortium (ICGC). They not only broadened cancer list genes but further identified novel dysregulated cellular processes, namely those engaged in chromatin regulation and epigenomic control and those involved in RNA splicing, metabolism, lineage maturation, and protein homeostasis (15).

It is well known that the genetic diversity among African populations is the most high and, therefore, its study requires a greater number of variants in order to determine the same amount of variation as in European ancestry groups, to do this, a larger sample size is required (16). TCGA project, which aims to uncover the main genomic alterations that cause cancer and construct a complete “atlas” of cancer genomic profiles (17), is targeting a large cohort of 11 122 patients involving 33 cancer types from 27 primary sites (18). TCGA, due to its cohort size, is considered to be one of the greatest projects with numerous samples, multidimensional genomic profiles, and thorough clinical information which are essential to detect the impact of genetic ancestry on genomic alterations. Despite these advantages, for *de novo* identification of genomic alterations specific to a racial group at a level specific to the type of cancer (18) and to capture even relatively common somatic mutations that are specific to those groups, the absolute number of samples of racial minorities like African ancestry groups in TCGA is still relatively small (19). Therefore, to better understand the genomic basis of the differences among all racial/ethnic groups, there is an increasing need to augment the number of underrepresented patients samples (18).

A large number of genomic variants were reported to be causally linked to or associated with a higher risk for various types of cancer. For example, in 11 members of two families of Greek origin, Karageorgos et al. introduced a NGS method for classifying all genetic variants with the propensity for family members to be predisposed to cancer. A total of 571 variants were reported in cross-comparison with data from the Human Gene Mutation Database, 47 percent of which were disease-related polymorphisms, whilst 26 percent were disease-related polymorphisms with further functional data, and 19% were functional polymorphisms. However, with some residual confusion as to their pathological importance, 4% were mutations causing putative diseases and 3% were mutations causing disease (20).

Laryngeal cancer is known to affect African-Americans more than European-Americans. In order to distinguish between environmental and ancestrally-inherited factors, Ramakodi et al. studied the genome-wide somatic point mutations from the tumors of a cohort including 57 European Americans and African Americans patients from TCGA. Differences between the two population in the distributions of the number of somatic point mutations per sample (the number of mutations varied from 29 to 313 with a mean of 151.31 for African-Americans and the number of mutations ranged from 46 to 1,026 with a mean of 277.63 for European-Americans) and the prevalence of context nucleotide signatures for somatic point mutations (C >G and C >A) were found. These nucleotide signatures in parallel with other factors may contribute to the variations observed in the mutation landscape between the two races. These findings suggest that the race, at the molecular level, play a significant role in the progression of laryngeal cancer with ancestral genomic signatures and explain the origin of the differences observed between the two studies races (21).

Similarly, for the sake of determining the role of ethnic differences in clear cell renal cell carcinoma (ccRCC) somatic mutation rate and gene expression, a cohort of 419 white and 19 African American patients identified through TCGA clear cell kidney (KIRC) dataset was examined. The GSE25540 dataset comprising 125 white and 10 African American patients was utilized for validation. The results showed that African American compared to white patients were enriched in the clear cell type B (ccB) molecular subtype that has worse prognosis and were significantly less susceptible to have Von Hippel-Lindau (VHL) mutations. Equally, in African American, the RNA expression disclose relative down-regulation of hypoxia-inducible factor and vascular endothelial growth factor -associated pathways. The outcomes of this work suggest that the genomic differences observed between African American and white ccRCC patients could be involved in the worse survival of African American patients (22).

The second most frequent malignancy in men worldwide is prostate cancer with 1,276,106 new cases and 358,989 deaths in 2018. When compared to white men, the incidence rates of prostate cancer in African American are higher with 158.3 new cases per 100,000 men and their death rate twice that of white men (23). The higher incidence and mortality of prostate cancer (CaP) observed in men of African Ancestry (AA) compared to men of predominantly European Ancestry (EA), may be due to genomic factors. To investigate this theory, the authors evaluated genomic profiles from the TCGA CaP cohort (n = 498) and analyzed the data from only 61 AA and 414 EA cases. Considerable differences were spotted by ancestry in the frequency of Transmembrane Serine Protease 2- ETS related gene (TMPRSS2-ERG) fusions (29.3% AA vs. 39.6% EA), speckle-type POZ protein (SPOP) mutations (20.3% AA vs. 10.0% EA), and Phosphatase and Tensin (PTEN) deletions/losses (11.5% AA vs. 30.2% EA). Differentially expressed genes (DEGs) between AAs and EAs demonstrated significant enrichment for prostate eQTL target genes. Enrichment of highly expressed DEGs for immune pathways has been observed in AA and for PTEN/Phosphatidylinositol 3-kinase (PI3K) signaling in EA. These results, through both genomic and transcriptomic analysis, indicated that the differences found may be biological contributors to racial discrepancies in the incidence and consequences of CaP (24).

Likewise, in order to highlight the genomic alterations linked to race, Koga et al. compared the frequencies of somatic alterations in a cohort comprising AA and AE prostate cancer patients. Mutations in Zinc finger homeobox 3 (ZFHX3), focal deletions in ETS Variant Transcription Factor 3 (ETV3), c-myc (MYC) ampliﬁcations in metastatic PCa, Histone-lysine N-methyltransferase 2D (KMT2D) truncations and Cyclin D1 (CCND1) ampliﬁcations in primary PCa were more frequent in tumors from AA patients. While rearrangements in Transmembrane protease, serine 2 (TMPRSS2-ERG) and deletions in PTEN were less frequent in AA compared with EA patients. In contrast, tumor mutation burden, microsatellite instability (MSI) status, and genomic alterations in select DNA repair genes, Cyclin Dependent Kinase 12 (CDK12), and in Androgen receptor (AR), which are the genomic features that could influence the clinical decisions, were found not to differ signiﬁcantly between the two groups studied. Despite the results indicating genomic disparities amongst AA and EA, the similarities found in the frequencies of genomic alterations in PCa therapeutic targets, suggest that precision medicine strategies could be evenly useful if applied fairly (25).

In another study, in order to perform deep sequencing of complete mitochondrial genomes in prostate cancer, McCrow et al. analyzed 87 tissue samples extracted from South African men with matched blood and prostate (77 with an African origin). Clinical presentation was skewed towards severe illness and contrasted either with or without benign prostatic hyperplasia to men without prostate cancer. One hundred forty-four somatic mitochondrial DNA (mtDNA) single nucleotide variants (SNVs) were identified, of these, 80 were found in 39 men with severe illnesses. Higher pathological stages were correlated with the number of somatic mtDNA SNVs and their frequency. Similarly, in men of African descent, the authors equate mutational load with the aggressive status of prostate cancer (26).

Abbad et al. indicated that the majority of genetic studies regarding African Breast Cancer (BC) remain restricted to studying BRCA1 and BRCA2 genes and their mutation spectrum variations. Thus, by collecting pertinent data from 43 studies in Africa depending on the following features: case control research, and the association of genetic variants with BC risk. Data on mutations and BC-related polymorphisms were given without setting a particular time. This research had omitted case-only studies and clinical trials. Therefore, to guide precise and more appropriate treatment interventions for the people of Africa, African scientists should be encouraged to identify more genes associated with BC employing high throughput methods such as NGS (27).

For Africa, Jaratlerdsiri et al. conducted the first tumor-normal paired genome sequencing. They registered for 15 cases a 1.8-fold rise in minor somatic variants in tumors of African and European origin, except one single hyper-mutated tumor with 55 mutations per mega base. In addition, they found a rise in oncogenic driver mutations in African tumors; approximately 30 percent of the affected genes were described for the first time in prostate cancer, and 79 percent of reoccurring tumorigenesis driver mutations emerged early. In African prostate tumors, complex genomic rearrangements were less frequent. Despite the fact that this research is preliminary, the findings indicate that further confirmation and analysis of the possible implications of increased mutational tumor load and tumor initiating gene alterations in clinically inauspicious prostate cancer will boost clinical outcomes in Africa (28).

It is important to point out that Sub-Saharan Africa’s (SSA) genomics research potential is relatively low and may hinder full benefits from genomics applications in medicine and clinical practice. About one-tenth of papers published on this genomics topic was related to non-communicable diseases where cancer present 6.1%. There are currently significant differences in genomics research ability among SSA countries and South Africa has the highest research performance in genomics, expressed in the investments made in its genomics and biotechnology activities (29). Challenges related to scarce resources affecting the implementation of genomics research in Africa include ill-equipped laboratories, lack of expertise, and enabling climate for local hospital research activities and inadequate connectivity to research centers. The research study challenges include comprehensive procedures, delayed funding, delays in building research units and inadequate human resource instruction, language difficulties and underestimation of cultural rules (30).

Several new major ventures, including the Human Heredity and Health in Africa (H3Africa) initiative, resolve a couple of the aforementioned barriers towards the establishment of precision and personalized medicine in African countries (31). The H3Africa project was built up to drive new genetic and environmental aspects forward on an African-relevant human diseases basis, as well as create resources for genomic research on the continent. For more than 70,000 members across the continent, this consortium jointly collects samples and data, followed by detailed clinical data on a range of communicable and non-communicable diseases. The consortium also invested substantial resources in the establishment of advanced African biorepositories, a bioinformatics network together with a prominent educational and training programs that drew up genomic data analysis skills and interpretation among bioinformaticians, health-care professionals and wet-lab researchers (32, 33).

## Cancer Epigenomics

In addition to genetic modifications, mutations, and polymorphisms, environmental factors also influence carcinogenesis through epigenetic changes. Epigenetics are heritable gene expression modifications that happen without altering the DNA sequences (34). Chemical elements are added to nucleotides and can regulate the expression of the surrounding gene(s). The epigenome concerns all of the chemical elements that have been attached to the entirety of an individual genome as a strategy to control the activity of all that genome components including genes. These epigenetic modifications cover two primary categories: methylation of DNA and modifications of histones. DNA methylation at the cytosine site of the 5th carbon typically occurs on CpG (CpG dinucleotide rich regions) islands present at the promoter and the proximal first exon of genes (35). Abnormal epigenetic pathways lead to the development of various diseases, including cancer. The aberrations found in the DNA methylation of human cancer could be assumed to fit into either of two types: transcriptional repression of tumor suppressor genes through the CpG Island promoter of hypermethylation and an extensive genome wide hypomethylation. In nearly every human malignancy, global DNA hypomethylation has been recorded (36).

One of the most prevalent kidney cancers is Renal Cell Carcinoma (RCC) (90% cases) with clear cell RCC (ccRCC) being the most common histological form (70% RCC cases). For unknown and unclear reasons, the incidence rates of ccRCC are higher amongst African American than European American. To reveal the causes of these differences, the authors performed a comparative integrative genomic and transcriptomic analysis on 50 AA and 266 EA. The findings of the differential methylation analysis showed 2,048 genes significantly varied by race. These genes have been found to be implicated in biologic processes, various molecular functions, and cellular component localization. Additionally, through the analysis of differential gene expression, 3,296 genes were found to be altered in AA compared with EA race. This work indicates that DNA methylation and mRNA expression are involved in tumor biology dissimilarities observed between AA and EA with kidney cancer (37).

Rubicz et al. carried out a study on a cohort of 76 African American men patients with prostate cancer to investigate if clinical manifestations of a more aggressive disease at diagnosis and prostate cancer recurrence are related to differential DNA methylation. Long-term monitoring detected recurrence of prostate cancer in 19 patients. Additionally, patients with cancer recurrence compared to patients without recurrence, were characterized by 23 differentially methylated CpGs. Methylation differences were also highlighted between regional vs. local pathological stage, men with metastatic-lethal prostate cancer vs. no recurrence, and higher vs. lower tumor aggressiveness. These findings show that prostate cancer aggressiveness observed in tumor tissues of African American patients, may be due to differentially methylated CpG sites (38).

Nieminen et al. characterized 69 sporadic Egyptian colorectal cancers for promoter methylation at 24 tumor suppressor genes, microsatellite instability, expression of mismatch repair, p53, and beta catenin proteins. Data were compared with 80 sporadic and familial Finnish colorectal cancers. The results indicated that Egyptian colorectal carcinoma significantly marked by elevated methylation of the microsatellite stable tumors as reflected by the average number of methylated genes per case and by the tumor suppressor gene methylator phenotype which was defined as methylation of 5 or more genes. Compared with these Egyptian samples, sporadic western, namely Finnish, cancers were characterized by a lower rate of methylation. Four genes are distinctly methylated between Egyptian and Western cases, wherein the relation in cyclin-dependent kinase inhibitor 2B (CDKN2B)/p15 to Egyptian roots was noteworthy. These results illustrate the potential impact of environmental exposures through DNA methylation in carcinogenesis (39). Another Abdulkareem et al. research showed different patterns of DNA methylation between Africans and European patients with colorectal cancer. Genome wide DNA methylation of 480,000 CpG sites revealed 4,103 of distinctively methylated sites between the two races, with 92% of CpGs (over 1,986 genes) being mainly methylated in Africans contrasted with 8% (246 genes) in European (40). As with all aspects of cancer omics, epigenetics in sub-Saharan Africa is poorly explored in cancer as in other non-communicable diseases (34).

## Cancer Transcriptomics

Transcriptomics is the analysis of RNA molecules on a wide scope, using high-throughput techniques, namely microarrays or RNA-seq. It explores the abundance and composition of a cell transcriptome (41). Transcriptomics helps us to view the genome’s functional elements and expose the global gene expression profiles associated with the disease (42). Transcriptome research is widely supported for the identification of biomarkers, precision medicine and investigation of biological and functional processes involved in health condition as well as in disease state such cancer (43). In a study conducted by Bernard et al. single cell transcriptomes analysis indicated the possibility of achieving high-resolution profiling of transcriptomic fluctuation occurring during multiphase progression of cystic pancreatic ductal adenocarcinoma precursors to pancreatic cancer (44). In addition to metabolomics, using a transcriptomic approach in another cervical cancer research, the authors assessed genes in 7 substantially enriched pathways, of which 117 differentially articulated genes appeared to be essentially involved in catalytic action. These findings suggested that both transcriptomic and metabolomic variables were associated with cervical cancer (45). In a study interested in non-small cell lung cancer (NSCLC), researchers performed a transcriptomic study of 1,027 NSCLC patients and 108 neighboring peritumoral tissues obtained from TCGA resource. This work revealed 2,202 genes presenting significantly diverse expressions in cancer cells in contrast with healthy controls (42).

To investigate the influence of racial variance in gene and miRNA expression on the biology of lung tumors with clinical relevance in African Americans (AA) and European Americans (EA), Mitchell et al. performed a comparative molecular profile on normal tissue and lung tumor samples, from AA and EA, using mRNA (n = 22 AA and 19 EA) and miRNA (n = 42 AA and 55 EA) expression arrays. The results of this study demonstrated that differential gene expression in EA lung tumors has been mostly affecting cell proliferation pathways. Whereas, the differential gene expression enriched in AA concerned stem cell and invasion pathways. Population-specific gene expression was in part determined by population-specific miRNA expression profiles. This comparative transcriptomic profiling highlighted intelligible distinctions between AA and EA in lung tumor biology (46).

Furthermore, Paredes et al. conduct a study to investigate the contribution of tumor immunology in the disparities observed between AA and Caucasian Americans (CA) populations. The authors performed a whole transcriptome sequencing to inspect the tumor and non-tumor adjacent tissues gene expression of AA and CA colon cancer patients. Additionally, as a validation cohort, they used the TCGA database from AA and CA. AA tumor samples present significant fold-change elevation in gene expression compared with CA for Interleukin 8 (IL8), forkhead box P3 (FOXP3), and Interleukin 1 beta (IL1B) genes. On the other hand, excessive gene expression of markers related to antitumor activity such as Interferon Gamma (INFG), Granzyme B (GZMB), and the immunotherapy targets Cytotoxic T-lymphocyte associated protein 4 (CTLA4) and Programmed death-ligand-1 (PDL1) proteins was observed in CA patients. Regarding the study of immune cell populations, the results showed that AA when compared to CA has an elevated number of mast cells, exhausted CD8+ cells and augmented T regulatory cells. Moreover, the differences between the two groups studied were also evident in the patterns of cytokine production in plasma. This work indicated the dissimilarities in colon cancer immune characteristics between AA and CA that may be implicated in insufficiency of proper immune defense mechanisms (47).

Esophageal cancer (EC), which is the seventh leading cause of cancer-related deaths, is a malignant tumor in the epithelial cells filling the esophagus. EC is accountable for over 400,000 deaths each year (48). Of all the cases of EC diagnosed globally, Esophageal Squamous Cell Carcinoma (ESCC) represents about 90% of the 456,000 incident esophageal cancers each year (49), and among them, around 80% take place in low-income regions of Asia and Africa (50). In sub-Saharan Africa (SSA) regions, ESCC is widely spread and considered as the third leading cancer. In Malawi, 59 patients with ESCC were reported by Liu et al. as a whole-exome tumor/normal sequencing and RNA transcriptome analysis. Based on the study of the genome transcription, ESCC may be divided into three different subgroups, which were distinguished by their cell cycle expression and the neuronal transcripts. The findings of the study revealed distinctive subtypes of ESCC in SSA and concluded that the endemic existence of this disease reflects exposure to carcinogens different from oncogenic viruses and tobacco (51).

In addition, the most prevalent pediatric cancer in equatorial Africa with endemic malaria is the Endemic Burkitt lymphoma (eBL) which almost constantly comprises the Epstein-Barrvirus (EBV), different from sporadic Burkitt lymphoma (sBL) characterized by decreased incidence in developed countries. For the purpose of understanding pathogenesis, Kaymaz et al. performed transcriptomic analysis using RNA sequencing from several primary eBL tumors versus Burkitt lymphoma (BL) tumors. Based on EBV genome type, in-hospital survival rates, anatomical presentation site, and suggesting that eBL tumors are homogeneous without marked subtypes, low expression distinctions were found within eBL tumors. The remarkably reduced expression of key genes in the immunoproteasome complex in eBL tumors carrying type 2 EBV compared with type 1 EBV is the salient difference revealed using surrogate variable analysis. In this study, the main part of pathway and expression differences was associated with PTEN/phosphoinositide 3-kinase (PI3K)/mechanistic target of rapamycin (mTOR) signaling pathway and was robustly compatible with EBV status rather than geographic specification. Moreover, a group of novel genes mutated in BL, including the coding gene for MutS Homolog 6 (MSH6), phospholipase C gamma 2 (PLCG2), Protein Kinase, DNA-Activated Catalytic Subunit (PRKDC), Regulation of Nuclear Pre-MRNA Domain Containing 2 (RPRD2), DNA repair protein (RAD50), Transcription factor activating enhancer binding protein 4 (TFAP4), BAF Chromatin Remodeling Complex Subunit (BCL7A), Proline Rich Coiled-Coil 2C (PRRC2C), and Forkhead box protein O1 (FOXO1) have been distinguished. Generally speaking, the data of this work demonstrated that EBV, in particular type 1, catalyzes BL tumor formation, reducing the requirement for certain specific mutations from the human genome (52).

Increasing transcriptomic innovations are nowadays recurring in order to diagnose cancer faster and more reliably, giving better prediction and prognostic value to cancer medical specialists and patients. Modern technologies like sequencing of RNA may replace existing imaging techniques to furnish further precise analysis of the transcriptoma and the aberrant expression that induces oncogenesis. Transcriptomics is used for the diagnosis of different cancer types for instance breast cancer, colorectal cancer, lung cancer, prostate cancer, and other tumors of unknown origin (53). Nevertheless, cancer transcriptomics and postgenomic medicine demand bioinformatics innovation and a critical review of the existing algorithm’s performance. Even so, due to interdependencies within gene entries, this analysis frequently faces considerable difficulty (43). Despite the importance currently given to cancer transcriptomics, the application of this approach in the African continent is still very poor compared to developed countries.

## Cancer Proteomics

The proteomics domain deals with the detection of the complete peptide and protein complement produced in an organism, tissue, or a cell and can be, in theory, more specifically linked to phenotypic modifications related to the pathogenesis of a certain disease. Proteomic studies may describe the functional situation of protein activities, protein-protein as well as protein-ligand interactions (54). Unlike transcriptomics, proteomics methods take the post-transcription, translation, and post-translational changes of polypeptides into account (55). In cancer, proteomic analysis can be used to follow disease development, to potentially distinguish markers for cancer diagnosis, and to characterize therapeutic targets on a body wide scale (56).

Urine and blood are both very promising sources of preclinical biomarkers for prostate cancer (PCa) in Africa. Contrary to African American populations, there is a lack of PCa proteomics research on indigenous peoples of African descent. Although several potential preclinical biomarkers of PCa were disclosed in Western studies, a limited number of studies in Africa have discovered and validated new possible PCa biomarkers (57). The study carried out by Adeola et al. on multi-ethnic cohorts of South African patients discovered novel candidate urinary protein biomarkers for prostate cancer. Throughout this study, proteomic analysis was performed based on mass spectrometry of pooled individual PCa samples, benign prostatic gland enlargement, normal healthy prostate samples, as well as patients carrying other uropathies to classify proteomic profile spectrum. A total of 1,102 and 5,595 protein groups and non-redundant peptides, respectively, were found in the pooling experiments. Twenty possible biomarkers in PCa were revealed and fold differences were spotted in 17 proteins. The analysis of 45 individual samples generated 1,545 and 9,991 protein groups, and non-redundant peptides, respectively. Seventy-three protein groups were identified as potential PCa biomarkers along with some known putative PCa biomarkers and demonstrated ethnic patterns within the PCa cohort. The identification of useful biomarkers tailored to several races and the good understanding of interethnic distinctions in this studied cohort, has been achieved thanks to the distinct proteins with ethnic orientation. The revealed candidate biomarkers, in addition to the demonstration of ethnic trend, regularly differentiated between PCa, benign prostatic hyperplasia, patients with other uropathies, and normal healthy individuals (58)

Ovarian cancer is the seventh most common cancer among women. In 2018, 295,414 cases and 184,799 deaths due to ovarian cancer have been identified. The lack of access to suitable treatment may be the cause of the elevated mortality-to-incidence ratio among African women (59). Ovarian cancer is characterized by the uppermost rate of mortality of all gynecological cancers because of its tardy detection and ambiguous symptoms. Hence, promising new potential tools for ovarian cancer diagnosis are needed. Rizk et al. intended to find a characteristic pattern of plasma proteomes that could be used to detect epithelial ovarian cancer in Egyptian females, compared to benign ovarian masses and normal controls. They further aimed to distinguish amongst early and advanced ovarian cancer profiling of plasma proteins, and between extremely serious and non-serious histopathological forms. The findings showed a 21-peak plasma proteome profile differentiating patients with epithelial ovarian cancer from healthy individuals, whereas a 5-peak profile distinguished patients with epithelial ovarian cancer from those with benign ovarian masses. With a recognition capability of 88.3% and an overall cross validation of 70%, the profile of 20 peaks was developed to differentiate between early and late disease stages. Of these 20 peaks, 14 were overexpressed in early stage ovarian cancer patients (stages I and II), but not significantly. Whereas, 6 peaks were over-expressed in late stage ovarian cancer (stages III and IV) (60).

The proteomics field has developed tremendously over the past 10 years especially in Europe, North America, and Asia, whilst it comparatively remains quite poor in Africa. In South Africa, the introduction of proteomics research is recent and a small number of scientists use it as a routine approach. The main challenges facing the large application of proteomics are associated with the rarity of scientists, and technical support in biotechnology in general. The handful of proteomics-trained researchers prefer to move on to other unconnected occupations upon accomplishment of training, often even before their research is publishable or published (55).

## Cancer Metabolomics

Metabolomics is the new omics technique used for the investigation of the presence and the abundance of metabolites (low weight biomolecules) in body fluids and cells (54). Urine, tissue, and serum are the most common specimens compatible with metabolomics analysis. Through genomics and proteomics, the metabolome changes according to the individual’s physiological and pathological condition and the detection of particular metabolites provide a potentially useful insight towards pathogenetic disease mechanisms (54). Metabolomic research is currently the prevailing approach for early detection and precise medicine and it may also provide information from a metabolic point of view regarding the development of cancers (42). Therefore, the comparison of the metabolic profile alterations of cancer cells with those of normal cells can contribute to the discovery of metabolites that would trigger carcinogenesis (61). Yang et al. published a detailed metabolomics and transcriptomics study on the possible diagnostic implications of cervical cancer and its metabolic character profile. 62 metabolites varied between cervical cancer (CC) and standard controls, five of which were selected as candidate biomarkers for CC, and were able to pave the way for diagnosis and screening (45). The Combination of transcriptomics and metabolomics approaches has elevated the effective recognition of both important functional genes and metabolic pathways in lung cancer patients. In a study in which the authors made an untargeted metabolomics assessment of 142 patients with non-small cell lung cancer (NSCLC) and 159 safe controls; 35 reported metabolites significantly differentiated between NSCLC patients and healthy controls, of which 6 metabolites were selected as possible combination biomarkers for NSCLC. Like in the previous one, the findings of this study confirm that the discriminating metabolic biomarkers detected can be used for screening and diagnosis of NSCLC (42). Researchers combined transcriptomics and metabolomics in another study on human prostate cancer to compare 25 paired tumor and adjacent non-cancerous tissues. Further confirmation of the results has been performed in an expanded cohort of 51 PCa patients and 16 patients with benign prostatic hyperplasia. The findings showed many abnormally expressed pathways at both the metabolic and transcription levels, including metabolism of methionine and cysteine, metabolism of nicotinamide adenine dinucleotide, and hexosamine biosynthesis. The sphingosine metabolite has also shown capacity to distinguish prostate cancer from benign prostatic hyperplasia with high sensitivity and specificity (62).

Breast cancer is the most common cancer among women. In 2012, 1.67 million new cases and 324,000 deaths of breast cancer were identified worldwide. The incidence rate of breast cancer varies considerably among different regions of the world (27 per 100,000 in Middle Africa and East Asia and 92 per 100,000 in Northern America) with the knowledge that the highest age-standardized mortality rate around the world was recorded in Africa (63). Triple negative breast cancer (TNBC), which is more common in African Americans, is a cancer in which the expression of estrogen receptor (ER), progesterone receptor (PR), and human epidermal growth factor 2 receptor (HER2) is missing. In the study conducted by Kanaan et al. the authors, through a comprehensive gas chromatography (GC)-mass spectrometry (MS) and liquid chromatography (LC)/MS/MS-based and unbiased metabolomic analysis, addressed a molecular understanding to detect the differences between TNBC and ER(+) breast cancer. The analysis was carried out on a series of breast carcinomas from African-American patients. The results of the global metabolomic profiling of tumor tissues determined a total of 418 featured metabolites, out of which 133 were found to be different between ER (+) and TNBC tumors. In the TNBC when compared to ER(+) tumors, the distinct biochemical pathways affected included those reflecting general augmentations in energy metabolism and transmethylation. Moreover, high levels of biochemicals linked with increased proliferation, redox balance, and the recently proposed oncometabolites, sarcosine and 2-hydroxyglutarate were found in TNBC compared to ER(+) tumors. The outcomes of this study highlighted the possibility of discovering new treatments based on the distinctive metabolic characteristics of these tumors (64).

In a study conducted on an Egyptian cohort, researchers aimed to compare the levels of metabolites in sera of 49 patients with cirrhosis and 40 individuals with hepatocellular carcinoma (HCC). The use of ultraperformance liquid chromatography coupled with quadrupole time-of-flight mass spectrometer (UPLC-QTOF MS)-based metabolomics, supplies helpful insight into suitable computational methods and experimental design for the discovery of serum biomarkers. The findings allowed candidate cirrhotic controls. It is important to recall that this is the first MS-based metabolomics study conducted on Egyptian cohort in order to discover candidate metabolites that could be used to detect HCC early in cirrhosis patients (65).

Despite all these studies carried out on cancer patients and these interesting results, the oncology community not only in Africa but worldwide still lacks knowledge of metabolomics and is uncertain about its methodological methods, technological problems, and clinical applications (66).

## Cancer Metagenomics

Metagenomics has widened the potential in targeting the microbes responsible for causing different kinds of cancer (67). Metagenomics is a valuable strategy to characterize and classify microorganisms in their home environment. The identification, analysis, and targeting of microbial diversity in tissue samples from cancer patients have been revolutionized with the implementation of metagenomic approaches (67).

Colorectal cancer (CRC) is the third most deadly type of cancer in the United States. The estimates of 2016 showed 134,490 new colorectal cancer cases (70,820 in males and 63,670 in females) and 49,190 deaths (68). In sub-Saharan Africa, colorectal cancer has been estimated to be the fifth most prevalent malignancy, according to the International Agency for Research on Cancer and the American Cancer Society (69). GLOBOCAN’s estimates for several countries in sub-Saharan Africa vary considerably (9). In Gambia and Mozambique, the estimated age-standardized incidence rate (ASR) per 100,000 was identified to be the lowest (1.5 in men and 1.0 in women for Gambia; 1.5 and 1.0 for Mozambique). In contrast, the highest ASR was reported in South Africa (15.6 and 9.5), due to racial and ethnic diversity (70). Given that the third leading cause of death in Morocco is colorectal cancer, Allali et al. contrasted the stool microbiome of Moroccan healthy individuals with the one of CRC patients. They follow a 16S rRNA amplicon sequencing approach to characterize the microbiome diversity and richness of samples from 11 CRC patients and 12 healthy individuals. Results revealed that cancer samples had higher amounts of Firmicutes, explicitly Clostridia, and *Fusobacteria*, notably *Fusobacteria.* Whilst Bacteroidetes were enriched in healthy samples, especially the *Bacteroidia* class. In diseased patients, *Porphyromonas*, Clostridium, *Ruminococcus, Selenomonas*, and *Fusobacterium* were substantially overrepresented. Outcomes of this study have enabled the identification of bacterial taxa pertinent to the Moroccan population and call for broader research to raise population-driven therapeutic methods (71).

African men are exposed to increased risk of prostate disease and infection. Feng et al. assume that the high-risk manifestation of PCa in Africa and the observed ethnic difference in turn, at least in part, may be due to pathogenic microbes. In this study, the authors reveal the microbial composition within prostate tumor tissue from 22 patients by means of metagenomic analysis of host-derived whole-genome sequencing results. What is interesting about this study is that it divided patients by race. The research revealed 23 common genera of bacteria amongst African, Australian, and Chinese prostate tumor samples. In the African vs Australian samples, the authors have found a substantial increase in the diversity of bacterial species. With an excess of *Eubacterium* linked to host tumor hypermutation, prostate tissue samples from African patients seem enriched for *Escherichia* and *Acidovorax*, considering core human gut microbiota. The high tumor mutation load in African vs. non-African specimens together with the increasing bacterial composition and abundance, suggests that bacterially-driven carcinogenesis in the prostate microenvironment may lead to aggressive manifestation of the disease in Africa (72).

Micro-organisms cause a large percentage of cancers, so metagenomics studies may promote cancer research by recognizing the microbes that are involved in cancer genesis and progression. Therefore, coming studies are promoted to scout the microbes roles in other different forms of cancer (67). Once again, this is noteworthy the very few studies on the microbiome relationships with most cancer types in the continent.

## Challenges and Recommendations For Cancer Omics Development In Africa

Given the enormous encumbrance of cancer in Africa, healthcare strategies need to catch the most cost-effective and precise approaches to test and diagnose the disease at an early stage. Even though up to 80% of the cancer incidence is in low- and middle-income countries, it only benefits from about 5% of global cancer spending (7). This low investment in the field of cancer is reflected in the limited number of studies carried out in the continent, in particular, those using developed methods such as the omics strategies. **Table 1** summarizes African studies on cancer omics.

**Table 1:** List of African studies on cancer omics.

Type of cancer	Omics method	Year of publication	Country	Reference
**Prostate Cancer**	Genomics	2015	South Africa	(26)
**Prostate Cancer**	Genomics	2018	South Africa	(28)
**Colorectal Cancer**	Epigenomics	2016	Nigeria	(39)
**Esophageal Squamous Cell Carcinoma**	Transcriptomics	2017	Malawi	(51)
**Endemic Burkitt lymphoma**	Transcriptomics	2017	Kenya	(52)
**Prostate Cancer**	Proteomics	2015	South Africa	(58)
**Ovarian Cancer**	Proteomics	2019	Egypt	(60)
**Hepatocellular Carcinoma**	Metabolomics	2012	Egypt	(65)
**Colorectal Cancer**	Metagenomics	2018	Morocco	(70)
**Prostate Cancer**	Metagenomics	2019	South Africa	(71)

Precision medicine, also known as personalized medicine and individualized medicine (73), is a modern healthcare approach that aims to produce the accurate treatment at the proper dose and time based on the individual’s health, diet, lifestyle, family history of disease, and ethnicity (74). We must emphasize that the majority of studies aimed at revealing the molecular profile of cancer have been carried out in patients from high-income countries. As cancer has become a global burden and cancer medicine is progressively guided by molecular alterations in high-income settings, low-income settings can be left behind. Therefore, researchers, funders, and policymakers must increase their efforts internationally to allow cancer research to cover the entire world (75). The main challenges facing the precision medicine implementation in Africa are manifested in the deficiency of infrastructure, equipment, transport, funding, trained personnel in laboratory medicine and data sciences, and evidence to support the applicability of the clinical response (75). In order to overcome this shortage and make precision medicine a reality in Africa, further genomics research and data collection relevant to indigenous populations are needed (76). Additionally, there is an increased need to transform genomics knowledge into genetic tests, diagnostics, or improved dosing algorithm (76).

Data such as medical histories and genetic test data are the basis of sizable cohort studies and personalized medicine (77). In the case of big data, it is hard for an organization to analyze, manage, and extract value from it through traditional methods and systems due to its big volume, velocity, and diversity (78). With the huge advance of sequencing techniques in biomedical domain, tons of molecular sequencing and genome profiling data were generated. Extensive projects such as TCGA gathered large scale genomics data which are publicly accessible. These data sets supply criteria for method development and raising of big data analysis performance (77). In low and middle income countries (LMICs), such as African countries, the main challenge facing the use of big data in precision medicine is learning how to start generating and harnessing the value of sharing big data. Additionally, because of the restricted availability of patients’ data, scientists and epidemiologists carrying out research in these regions, may find limited use of big data. One of the significant challenges in using health big data is managing the transition from using paper to using electronic documents, especially with the fact that the clinicians still prefer to utilize paper and are less affected by the capabilities that the infrastructure provides for sharing data through information system exchange. We must point out that the infrastructure needed to implement big data initiatives is generally sophisticated because it encompasses many technology platforms, data types, and stakeholders (78). For these reasons, big data initiatives should be encouraged by the ministries of health and research. In order to ensure greater benefits, the ministries must also construct efforts in an open and public framework and include public-private partnerships. Moreover, to link data with practice, ministries should establish relationships with physicians and data scientists. In this context, in order to provide an extensive infrastructure that can lead, in the health sector, to the production and use of big data, as example to follow, the Rwandan government has proposed the Rwanda Health Information Exchange (RHIE) initiative. RHIE strives to continuously collect and assemble health data and encourage service providers, organizational decision makers, and patients to reuse it (78). Generally, LMICs are rapidly beginning to generate data that has become “big” in nature, especially with the widespread and growing prevalence of cloud infrastructure, web-based technologies, mobile devices, and other technologies (78).

The emergence of high-performance omics-based technology has illustrated the demand for computational biology, and also, state-of-the-art experimental biospecimen banking. Inappropriate biological specimen documentation and storage can lead to distorted biochemical inferences, histopathological examination, and expected therapy. A suitable biorepository specimen, associated with pertinent data for research purposes and following rules of relevant ethics, policies and processes, is, therefore, essential infrastructure component for the development of personalized medicine based on high-throughput omics in Africa. Many revolutionary genomics projects including the Human Genome Project (HGP), the Human Proteome Project (HPP), and TCGA have gained greatly from biorepositories of specimens (79). In Africa, however, it is evident that there is restricted scope for biobanking, and that processes such as fresh snap-frozen tissue sampling are not easy to be conducted in the majority of the parts of the continent because liquid nitrogen is mainly out of control (57).

Regarding cancer genomics research capacity in Africa and mainly in East Africa, only Kenya and Sudan have the maximum capacity to carry out research into cancer genomics. Both countries have academic facilities fitted with state-of-the-art labs. Biosciences Eastern and Central Africa Hub Genomics and Bioinformatics Platform in Kenya is equipped with capillary and second-generation sequencing facilities that will enable East African researchers to conduct genotyping activities and sequencing for genomes and metagenomes (80).

African authorities should concentrate on financing facilities, researchers, and support for scientific training and with favorable improvements in health policy, molecular methods based on omics should be incorporated into routine clinical practice (7). Furthermore, to exploit the advantages of bioinformatics and data science in cancer omics research in Africa, the first step is to overcome the issue of limited skills in bioinformatics and genomics all over the continent. Adequate computational infrastructure, teaching laboratories, availability of training spaces, server systems, and social and political stability are some of the factors influencing the organization of sustainable training programs. Across Africa, several bioinformatic training initiatives have been launched such as the doctoral training in bioinformatics provided in Uganda and Botswana through the Collaborative African Genomics Network (CAfGEN) (81), the Eastern Africa Network of Bioinformatics Training (EANBiT)  (81) which supplies bioinformatics training in Kenya as part of a M.Sc. program in bioinformatics, and the African Genomic Center (TAGC) launched in Cape Town, South Africa in 2018 which comprises a powerful bioinformatics training component (81), not to mention the H3 African Bioinformatics Network (h3ABioNet) (82) that, in different countries, organize training programs aimed at enhancing the computational skills of biology and health scientists in Africa (81) (83). In addition, African scientists, regardless of their location, can be trained through online training programs, workshops on bioinformatics organized by world leading scientific organizations, short courses, and complete online degree programs established by some African universities (83). The current efforts in Africa to improve training opportunities in Bioinformatics and Genomics are expected to generate scientific experts to drive the prosperity of genetic and genomic research in Africa (83). In this regard, to improve skills in medical genetics and genomics, key healthcare personnel must be involved. For this purpose, training of the healthcare staff and clinical researchers in genomic medicine, through professional development courses, is the foundation of efficacious adoption of genetic and genomic evidence into clinical cancer application. Generally speaking, training initiatives in genomic medicine domain are in their infancy, but the African continent confront further challenges at the institutional and logistical levels. To achieve the objective of developing knowledge and capacity in genomic medicine, during a common conference of the African Society of Human Genetics and the US National Health Institutes (NIH)-funded H3Africa Consortium in 2016, Senegal, the participants launched The African Genomic Medicine Training (AGMT) Initiative (84). Healthcare staff like doctors, pharmacists, nurses, who are not geneticists, are the main beneficiaries from this durable genomic medicine training initiative. This approach provided graduate and postgraduate programs, short courses as well as public engagement activities. The AGMT initiative also gives the opportunity to patients who wish to be advocates in the fields of genetics and genomics to participate in the courses. We must point out that, across Africa, AGMT was the first extensive community training initiative in genomic medicine (84).

Regardless of the fact that bioinformatics typically needs much less infrastructure investment compared to science-intensive disciplines, basic equipment like robust computer systems, access to basic databases and software, dependable source of electricity, high-speed Internet are essential. Fortunately, the challenge of the lack of internet and computers is gradually disappearing in Africa allowing research to progress. Moreover, building research centers well-equipped with bioinformatics resources and integrating specific departments in bioinformatics within the existing institutes is improving training conditions (83). Furthermore, to help Africa mitigate some of the infrastructure hurdles, the H3ABioNet project has participated in the renovation of several training laboratories and the provision of servers and computers within the network in Africa. In order to allow geographically different classrooms to take part in a live and interactive training workshop and to deal with the wide geographical distances, in the continent, live video streaming services such as Vidyo are used (85).

## The Way Forward in Omics for Africa

There is an intensified optimism about the role omics may help in addressing general health disparities, particularly after the accomplishment of the human genome project and the ongoing African genome sequencing. From this, we can say that there is an increased need for genomics research alliances in Africa to carry out omics studies and to reveal means in which the results of these research could be practically incorporated into health care for the interest of African populations (86). For all these reasons we propose the immediate establishment of an “African Cancer Genomics Consortium”. Our call to this all-omics initiative aims to raise the efforts in order to minimize the massive negative effect of this fatal disease on an already brittle continent and to encourage others to become concerned. For example, studying African genomic variation constitutes the next frontier of genetic medicine, it will therefore be necessary to develop an African genomics workforce to implement such broad research in cancer. These efforts can build on the foundation of many successful initiatives such as H3Africa (87).

## Conclusion

As mentioned earlier, compared to non-African populations, the genetic diversity of the African race is the highest. Therefore, comparative studies conducted on ethnically diverse populations, mainly in Africa, are crucial to investigate the genetic basis of complex disease and phenotypic adaptation. In Addition to phenotypic details about various traits such as disease likelihood and response to drug, the comprehension of levels and patterns of difference in African genomes, will be pivotal to highlight the genetic basis of environmental adaptation and to discover new and effectual therapeutic treatments for disease (88). In the case of cancer, racial dissimilarities observed in the mortality and the morbidity of the illness, could be minimized through an understanding of contributing genetic factors. This objective can be attained by studies conducted, for example, on African American participants (89). In the field of cancer omics, the USA is the country with the most efforts to apply these approaches to African immigrant populations (**Table 2**).

**Table 2:** List of studies on cancer omics carried out on migrant African.

Type of cancer	Omics method	Year of publication	Country	Reference
**Laryngeal cancer**	Genomics	2016	USA	(21)
**Clear cell renal cell carcinoma**	Genomics	2016	USA	(22)
**Prostate cancer**	Genomics/Transcriptomics	2020	USA	(24)
**Prostate cancer**	Genomics	2020	USA	(25)
**Clear cell renal cell carcinoma**	Epigenomics/Transcriptomics	2020	USA	(37)
**Prostate cancer**	Epigenomics	2019	USA	(38)
**Non-small cell lung cancer**	Transcriptomics	2017	USA	(46)
**Colon cancer**	Transcriptomics	2020	USA	(47)
**Triple negative breast cancer**	Metabolomics	2014	USA	(64)

The world is expecting massive data/information generation at most cancer levels in the genomics, transcriptomics, proteomics, metabolomics, epigenomics, metagenomics, etc. The translation of these data into practical clinical ways (i.e., identifying pathways/pathophysiology) will require considerable work in the coming years. To do this, we need integration of system biology approaches, emerging technology, and new computational and mathematical methods for in-depth research into cancer.

As technologies and strategies in Omics are continually evolving, the emerging technique single cell sequencing offers a valuable tool to enhance our knowledge of tumor cell heterogeneity in order to guide tailored cancer treatments. Moreover, this new technique is a way of distinguishing subpopulations of cancer cells in a single patient. Single cell sequence analysis can tend to be crucial to comprehend the etiology, development, and drug resistance of cancer countries (90). Although the single cell genomics is its infancy, African cancer research is called to engage in this very promising trends for research in cancer.

Being a continent predominantly populated by low and middle-income countries and highly impacted by cancer, various obstacles have been identified working against the common implementation of these strategies in Africa. There is an urgent need to expand country- or regional-based cancer research initiatives and collaborate with partners inside and outside the continent to overcome these limitations. African governments should also be involved in the implementation of cancer omics strategies by providing a useful and sustainable research environment in local government-owned institutions that will provide researchers with many opportunities to build their capacity in bioinformatics and omics through training programs. In this context, it is reassuring that many initiatives and projects have been put in place in different African countries (81), but additional efforts should be made to generalize these approaches on the continent. In addition, the hurdle of the limited number of studies conducted on African populations must be overcome, and research should be encouraged and pushed towards the detection of omics (genomics, transcriptomics, proteomics, metabolomics, epigenomics, and metagenomics) alterations in the case of cancer in a highly genetically diverse population such as the African one. Collaborative research geared towards the investigation of these cancer omics in African patients must also be motivated, both at continental level and with international partners. The prospected “African Cancer Genomics Consortium” would be mandated to promote such collaborative projects and engage in research activities for cancer precision medicine.

## Author Contributions

IE wrote the manuscript. IA conceived the study and wrote the manuscript. SS wrote the manuscript. KO conceived and designed the study, and reviewed the writing of the manuscript. SH and NA reviewed the writing of the manuscript. CN and SA conceived and designed the study. YB conceived the study and reviewed the writing of the manuscript. HG conceived and designed the study and and wrote and reviewed the writing. All authors contributed to the article and approved the submitted version.

## Conflict of Interest

The authors declare that the research was conducted in the absence of any commercial or financial relationships that could be construed as a potential conflict of interest.
